# Heterogeneous Risk Perceptions: The Case of Poultry Meat Purchase Intentions in Finland

**DOI:** 10.3390/ijerph10104925

**Published:** 2013-10-11

**Authors:** Jaakko Heikkilä, Eija Pouta, Sari Forsman-Hugg, Johanna Mäkelä

**Affiliations:** 1 MTT Economic Research, Latokartanonkaari 9, Helsinki FI-00790, Finland; E-Mails: eija.pouta@mtt.fi (E.P.); sari.forsman-hugg@mtt.fi (S.F.-H.); 2 Department of Teacher Education, University of Helsinki, P.O. Box 8 (Siltavuorenpenger 10), Helsinki FI-00014, Finland; E-Mail: johanna.m.makela@helsinki.fi

**Keywords:** poultry, consumer behaviour, risk, food safety, consumer heterogeneity

## Abstract

This study focused on the heterogeneity of consumer reactions, measured through poultry meat purchase intentions, when facing three cases of risk. The heterogeneity was analysed by latent class logistic regression that included all three risk cases. Approximately 60% of the respondents belonged to the group of production risk avoiders, in which the intention to purchase risk food was significantly lower than in the second group of risk neutrals. In addition to socio-demographic variables, the purchase intentions were statistically associated with several attitude-based variables. We highlighted some policy implications of the heterogeneity. Overall, the study demonstrated that risk matters to consumers, not all risk is equal, and consumer types react somewhat differently to different types of risk.

## 1. Introduction and Background

Consumer behavioural responses to risks have been studied in relation to consumer food choices, as well as by focusing on purchase intentions under individual food risk types. Typically, these studies have focused on either production-related risks such as hormone treatment [1,2], chemical control substances [3] and genetic modification (GM) [4,5,6,7], or on animal disease risks such as *Campylobacter* [8], *Salmonella* [9,10] and bovine spongiform encephalopathy (BSE) [11,12,13]. It has been recognised that the risks are subjectively perceived by consumers, even if objective information on probabilities and impacts is available. Consumers respond to risks based on their subjective perceptions of the probability of the event (*i.e.*, risk perceptions) and on their attitude towards the event (*i.e.*, general positive or negative predisposition) [14]. Additionally, consumer reactions depend on several factors that are risk type-specific, including the extent to which they can affect the risk themselves, how familiar they are with the risk, to what extent the exposure is voluntary, how severe the consequences are (irrespective of their probability), and so forth [15,16,17,18]. In this study, we focused on the heterogeneity of consumer reactions to a set of differing risk cases.

In previous studies, geographical differences among consumers have been documented as a source of heterogeneity. Consumers in the European Union (EU) have been particularly sceptical regarding genetically modified products (e.g., [15,19]), as well as hormone-treated beef [20], and the risk perception in general has been found to be higher in Europe than in North America or Asia [4]. Country-specific differences have also been documented [21,22]. The analysis of perceptions and attitudes toward risk has often focused on the differences between experts and the public [20,23,24,25]. However, socio-demographic factors such as age, gender, whether the respondent has children and whether the respondent lives in urban or rural surroundings have also been found to correlate with the risk response (see, e.g., [6,10,26,27,28]).

Beyond individual sources of heterogeneity, segmenting consumers could be useful in risk studies to assess how various types of risk affect heterogeneous consumer segments and their buying behaviour. Identifying the reactions towards specific risks in separate segments helps in targeting information and product development at those segments that are most sensitive [29]. In addition to the design of policies, understanding the behaviour of heterogeneous consumer segments is a necessity for the viable implementation of policies such as risk communication. Segmenting of the population according to information needs and the development of information with high levels of personal relevance to specific segments allows more effective communication of the risks [20]. Additionally, if consumer reactions to risk differ according to the segment, the benefits of precautionary policies will also differ among segments, suggesting that equity considerations are also involved. Therefore, segmentation appears to be a prominent approach in targeting risk management policies, communication and research.

However, only a few studies have reported consumer segments based on their food risk perceptions, attitudes or behavioural responses. In a review of consumer reactions to a food crisis, Wansink [30] formed theoretical consumer segments from a combination of risk perceptions and pre-existing attitudes towards risk. Varying the level of risk perception and attitude in a matrix of four fields, he illustrated the behavioural response to risk in these four segments. In an empirical, study Kennedy *et al*. [31] explored the content of food safety attitudes, which included components of concern, trust, the desire for a high level of regulation, acceptance of the number of suffering people, and preference for the right to purchase safe or unsafe food. Based on these attitude components, the authors identified five segments of consumers, with one of them (apprehensive consumers) benefiting the most from safety information. Focusing on Creutzfeldt-Jacob disease, Payne *et al*. [29] used behavioural, attitude and risk perception information in an experimental setting to segment consumers. They observed that group memberships depend on consumption levels, but demographic variables also provide profile information on the segments. These previous studies have segmented consumers based on their risk perceptions, risk attitudes or behavioural responses towards risk. However, none of these studies has focused on segmentation based on behavioural responses (*i.e.*, purchasing decisions) under various risk types.

There have also been a few studies comparing the reactions to various risk types among the general public [32,33,34], including the case of food risks [35,36,37,38,39]. However, most of these studies have focused on similarities and differences between risk types, not the heterogeneity of people in their reactions. This implies that in food safety issues the general profile of individuals sensitive to various risk types is unknown. In the case of food purchasing decisions, it would also be beneficial to identify those individuals who are particularly sensitive to any of the hazards.

In this study, the segments of consumers were formed based on differences in behavioural intentions, but they were identified according to risk-related attitudes and socio-demographic or geographic factors. Moreover, we simultaneously considered several types of risk. By recognizing that beliefs precede attitudes, which further affect purchasing intentions, we followed the ideas of Ajzen [9,40,41,42] in a simplified form, where the attitudes toward risk were assumed to precede purchasing behaviours. Instead of focusing on subjective norms, which in the food-buying settings are typically inside household norms, or on perceived behavioural control, which in the case of hypothetical risks is challenging to concretize in a survey, we focused on various types of attitudes that can be assumed to affect the buying intentions for poultry under various types of risk. Although there is extensive literature on attitudes related to food risks (for a review, see [43]), the content of attitude constructs applied to model subjective risk evaluations by consumers varies considerably [5,20].

We applied the segmentation concept to the behavioural reactions of Finnish consumers to various risks from poultry meat. The case of poultry is interesting for several reasons. The consumption of poultry meat in Finland has increased significantly in recent years. The increased demand has partly been covered by imports, meaning that international safety risks can rapidly enter the markets due to globalisation of the food chain. At the same time, consumers have become increasingly interested in how meat is produced and how safe it is. If safety is compromised, short- or long-term reductions in demand are to be expected, as demonstrated in Europe, for instance, by avian influenza H5N1 [44,45]. Furthermore, poultry meat can be associated with several risk types and thus fits the aims of the study. We presented the respondents with three different risk cases related to a biological hazard (increased risk of *Salmonella* in poultry), a chemical hazard (chemical treatment of poultry meat) and a technological hazard (use of genetically modified feed), and analysed how various perceived risks affect purchase intentions, and whether consumer segments exist that react uniformly to all of these risk cases. By analysing the role of attitude constructs such as health orientation, domestic preference, GM negativity and safety orientation, we also aimed to provide information on the characteristics of the segments.

## 2. Methods and Data

### 2.1. The Survey Data

Since safety features have no observable variation or direct price in stores, experiments [6] or surveys revealing purchase intentions need to be used to estimate their demand. Here, consumer survey data on poultry meat consumption were used. An online Internet survey conducted in November 2007 provided information on the reaction of consumers to risk types. The data were collected by sampling 2,500 respondents from the Internet panel of a private survey company, Taloustutkimus. The panel comprised 20,000 respondents who had volunteered to participate in the panel [46]. The otherwise random sample was balanced toward population by adding 70 respondents representing the category of older respondents. The consumer data set (N = 1,312), with a response rate of 51%, was a representative sample of Finnish Internet users between the ages of 18 and 79 years. The sample was representative of the general population regarding gender, age, income and geographical location (Table 1), but the educational level in the sample was somewhat higher and the proportion of individuals with children in the family somewhat lower than in the general population. In the data set, 95% of the consumers included poultry in their monthly diet and almost half of them perceived that the proportion of poultry had increased in their diet during the previous five years.

**Table 1:** Descriptive statistics for the respondents in the data set and the 18- to 79-year-old population in Finland.

	In data	In population *
Proportion of females, %	51	51
Mean age, years	49	47
Proportion of people with a higher educational level (college or university), %	38	26
Proportion of people living in households with a gross income under €40,000, %	42	42
Proportion of people with children (<18 years) in the family, %	29	42
Proportion of people living in northernmost Finland (Lapland), %	4	4
Proportion of consumers having poultry in their monthly diet, %	95	N/A
Proportion of consumers who have increased the share of poultry in their diet during the last five years, %	49	N/A

* Source: www.stat.fi, 2009. N/A denotes data not available.

For dependent variables, the survey measured the conditional purchase intentions of the respondents regarding poultry products under three different risk cases. These three cases are here collectively called “risk products”, but it should be noted that the last two cases are objectively only potentially riskier than standard products. A risk product here thus refers to risk as perceived by the consumer. Case 1 concerned biological risk, the zoonotic risk related to *Salmonella*, with a quantitative explanation. The levels of morbidity and mortality derived from the Finnish National *Salmonella* Control Programme were provided for the respondents. The respondents were told that 3,300 persons annually become mildly and 400 seriously ill (requiring attention from a doctor or hospital treatment) after eating poultry meat. They were asked to consider a situation where 19,800 persons become moderately ill, 2,400 seriously ill and additionally four persons die annually due to eating poultry meat. Half of the respondents, chosen randomly, were asked how the risk would affect their consumption decisions if the poultry were half of the current price. These respondents were provided a response scale of four alternatives for increased use (25%, 50%, 75%, 100% increase), one alternative of “no effect”, and four alternatives for decreased use (25%, 50%, 75%, no use). Half of the respondents were asked about their reactions if the price remained unchanged. For them, the response scale was “no effect” and four alternatives for decreased use (25%, 50%, 75%, no use).

Case 2 concerned chemical safety, and the respondents were presented a scenario where chemical treatment is an alternative approach to maintain product safety: “The safety of poultry meat in Finland is ensured with good production hygiene throughout the production chain. An alternative approach is to treat the meat products before they reach the consumer with chemicals to eliminate potential pathogens. International trade negotiations may lead to the market entry of chemically treated meat in the EU.” The exact name of the possible chemical (chlorine) was not mentioned in the survey. After this information, a four-level scale was presented to the respondents to indicate their willingness to choose the product: (a) if it was cheaper than the conventional product; (b) if it had the same price as the conventional product; (c) even if it was more expensive than the conventional product; or (d) would not choose the product at all.

Case 3 related to technological risk, and it was explained to the respondents that genetically modified soya was not at the time of the survey used in poultry production in Finland but was a future alternative (feed containing GM soy entered the Finnish feed markets in 2013). The purchase intention was asked if GM feed was used in production. The same scale was implemented as in Case 2.

In all three cases, the respondents who would still buy the product (although possibly at a lower price) were coded in the “yes” category, and the respondents who would stop buying altogether were coded in the “no” category. In other words, in Case 1, a decreased amount of use was also coded in the “yes” category.

We tested alternative modelling approaches. First, we used the whole range of scales of the dependent variables, *i.e.*, the original four-level scale for chemical risk and technological risk and the original five-level scale for biological risk. We undertook ordinal regression (ordered logit model). However, the test for parallel lines implied that the slope coefficients in the models were not equal across all response categories. For such data, the appropriate model would be the multinomial logit model instead of ordinal regression. We estimated the multinomial logit models, but in the case of the four-category dependent variable, no stable solution was found because of the low proportion of responses in one category. The model with a three-category dependent variable did not provide us with considerably more information than the binary approach, and because the multicategory approach made the comparisons between the risk types more difficult, we ended up using the binary coded variables, as detailed above.

To provide independent variables for modelling, the survey included questions relating to consumer patterns of using poultry meat, several attitude and belief questions relating to poultry production, and socioeconomic background variables. The relative amount of poultry meat in the diet was constructed from the five-class scale measuring the frequency of poultry consumption. The final variable was expressed relative to the consumption frequency of other types of food (vegetarian, fish, beef, pork).

The attitude variables included health orientation, domestic preference, GM negativity and safety orientation (Table 2). Structured focus group discussions [47] on poultry meat with different types of consumers were utilized in developing the measures. The salient issues from the focus groups were also reflected with measures available from the literature (e.g., [48,49,50,51]). The reliability of health orientation, domestic preference and GM negativity measures was analysed with Cronbach’s alpha (Table 2).

Health orientation was measured with eight statements from the measures of general health interest in the food setting presented by Roininen *et al*. [48] using a Likert scale from one (fully disagree) to five (fully agree). One of the items was dropped to obtain an acceptable Cronbach’s alpha and seven items formed the final variable, summarised by the mean of the item responses. The statements were as follows: (1) For me it is important that my daily food is low in fat; (2) For me it is important that my daily food contains plenty of vitamins and micronutrients; (3) The healthiness of snacks is irrelevant to me (reversed); (4) I do not avoid any foods, even if they raise my cholesterol level (reversed); (5) I make sure that all the food I eat is healthy; (6) I eat what I want and do not care much about the healthiness of food (reversed); (7) I always follow a healthy and balanced diet.

**Table 2:** Descriptive statistics for the poultry use variable and the attitude-based variables, including Cronbach’s alphas where applicable.

	Minimum	Maximum	Mean	Std. deviation	Cronbach’s alpha
Relative amount of poultry meat in the diet	0.26	2.27	0.81	0.26	
*Attitude-based variables*					
Health orientation	1.00	5.00	3.63	0.73	0.857
Domestic preference	1.17	5.00	4.17	0.64	0.786
GM negativity	1.00	5.00	3.94	1.03	0.931
Safety orientation	0.26	2.37	1.14	0.18	

Based on the focus groups and previous studies on country of origin effects [50,51], the attitude towards Finnish production was measured by six belief statements regarding domestic poultry production: (1) By buying Finnish poultry meat I can affect employment in Finland; (2) I buy Finnish poultry meat, because I value Finnish primary production; (3) I do not pay attention to the origin of the poultry meat (reversed); (4) Finnish poultry meat cannot be distinguished from imported poultry meat (reversed); (5) I buy Finnish poultry meat because I trust Finnish food production; (6) Finnish poultry products should be marked with a label to inform the consumers about the origin. A Likert scale from one (fully disagree) to five (fully agree) was used. The final variable of domestic preference was the mean of the responses to the six statements.

Instead of general attitudes towards GM (e.g., [49]), the GM negativity statement was targeted specifically at the use of GM feed in poultry production. The measure was formed as a mean from four statements measuring beliefs related to GM feed in the poultry production chain with a Likert scale from one (fully disagree) to five (fully agree): (1) The impacts of meat grown using GM feed on humans are not known; (2) The impacts of GM feed on production animals are not known; (3) The impacts of GM feed on the natural environment are not known; (4) The genetic modification of agricultural plants is ethically questionable.

The safety orientation was constructed from a question measuring the importance of several quality cues [52,53,54] faced when buying poultry products. In the final variable, the respondents’ rating of safety from one (not at all important) to five (extremely important), was divided by the mean of the importance of the other attributes: taste, healthiness, duration of use, price, easiness of use, attractive appearance, suitable package size, wide range of available products, and clearly marked country of origin. In other words, safety orientation measured the importance of safety relative to the importance of the other attributes.

The correlations between the attitude variables were relatively low, being under 0.3 between all the variables. Although low, the correlations were significant between domestic preference and all the other attitude variables, indicating a general trust in domestic food, as also reported in many previous studies.

The socio-demographic variables tested in the models were gender, age, education, income, families with children, and the residential region. Income and age were analysed in classes, as reported later in Table 5.

### 2.2. The Statistical Models

For each of the risk products, a model of purchase intention was constructed. As the dependent variables were dichotomous, the method used for modelling each purchase intention separately was logistic regression analysis [55]. The dependent variable in the models was whether the respondent would continue buying poultry meat under a change in its safety or stop buying it. The independent variables were selected based on the assumption of attitudes explaining behavioural intentions. We started the model specification with correlation analysis identifying mutually correlating independent variables and variables having a significant correlation with each dependent variable. The full set of alternative independent variables was used as a starting point for the model development. All non-significant variables (*p*-value over 0.10) were individually removed from the model, starting with those having the highest *p*-values. In this manner, the intended purchase was explained by the previously described attitude variables, but also by the socio-demographic variables. In addition, the relative amount of poultry in the diet was included in the model.

A few methods are available to study heterogeneity, but if there are no strong, theory-based assumptions regarding the sources of heterogeneity, segmentation through latent class models is a good method for the analysis. It does not require *a priori* assumptions regarding the sources of heterogeneity and deals well with multiple variables. The potential heterogeneity of the respondents regarding the purchase intentions was therefore analysed by latent class logistic regression for binary choices for all three risk products. It was also expected that latent class models would improve the explanatory power of the logit models. The basic assumption in a latent class model for binary choice data is that the parameters of the regression model differ across the estimated classes [56].

In the general case, the model specification is:

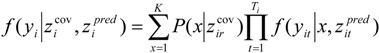
(1)


The dependent variable is a dummy variable for purchasing decisions under risk (*y_it_*). The indexes *i* and *t* refer to the individual and replication, *i.e.*, three purchasing decisions (one for each risk product) (*T* = 3). Behind the observed variables exists an unobserved nominal variable *x* that indicates *K* separate classes, each having their own distribution of observed variables, *y*. In the latent class model, prior assumptions of the reasons for heterogeneity are not needed. Instead, the attitudinal and socio-demographic variables *z^cov^* associated with the class membership are empirically examined. The index *r* indicates each background variable. *z^pred^* is a set of *Q* predictor variables influencing the dependent variable in each of the unobserved classes. In our case, the predictors were the case-specific dummy variables (Cases 1 and 2), while Case 3 (GM feed) was the reference level (*Q* = 2).

In the case of a binary dependent variable, the probability of *y* = 1 obtains a logistic form:

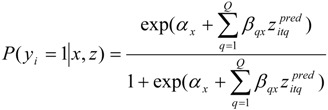
(2)
where α and β are model estimates. A detailed description of the methodology is available in Vermut and Magidson [57]. In estimation, the covariates (*i.e.*, attitude and socio-demographic variables) were active, thus affecting the solution, and they were selected in the model if the significance level of the coefficient was below 0.10. The model provides probabilities of class membership for each observation, and the observations were classified into the most probable class. The latent class model for binary choices in Latent Gold software was used to estimate the model. The Bayesian information criterion (BIC) indicated that the optimal model was a two-class model. Although the Akaike information criterion (AIC) continued to decrease, the improvement from the two-class model was minor (Table 3).

**Table 3:** Bayesian (BIC) and Akaike (AIC) information criteria for selecting the number of classes.

Number of classes	BIC(LL)	AIC(LL)	L²	R²
1	4,264	4,248	3,583	0.24
2	3,571	3,478	2,783	0.56
3	3,604	3,433	2,707	0.66
4	3,668	3,419	2,664	0.57
5	3,715	3,388	2,603	0.58
6	3,786	3,381	2,566	0.58

## 3. Results

The measures of purchase intentions revealed the reactions of the respondents to the three risk cases in poultry production. In Case 1 (biological risk), about half of the respondents stated that they would decrease their consumption of poultry if the risk of morbidity and mortality increased six-fold. In the case of no price impact, only 13% of the respondents would continue the use of poultry at the current level and 57% at a lower level. If the increased disease risk would lead to a price reduction of 50%, 20% of the respondents would continue to use poultry at the current level. Five per cent of the respondents were less sensitive to risk and would consider increasing the use of poultry if the product price decreased by half. However, the proportion of respondents not willing to buy was the same, 30%, regardless of the price effect (Table 4).

Nearly 90% of the respondents were of the opinion that they would not choose chemically treated poultry meat (Case 2). Seven per cent of the respondents, however, were willing to select the chemically treated product if it was cheaper than conventional meat.

**Table 4:** Distribution of buyers based on purchase intentions, proportion of buyers, and correlation between the purchase intentions under different types of risk.

	Distribution, % of respondents				Proportion of buyers %	Phi coefficient for correlation...	
						(*p*-value)	
	Would increase purchases *	No effect on purchases	Would decrease purchases	Would not purchase		...with Case 2	...with Case 3
Case 1	2.3	16.3	51.4	30.0	70.0	0.056	0.148
(Biological risk)							
						(0.043)	(0.000)
	Would purchase if cheaper than conventional	Would purchase if the same price as conventional	Would purchase even if more expensive than conventional	Would not purchase			
Case 2	7.5	2.9	0.7	88.9	11.1		0.361
(Chemical risk)							
							(0.000)
Case 3	23.8	12.0	1.0	63.2	36.8		
(GM-feed)							

* This option was available only for the first sub-sample, where it was described that the price would decrease by half. The column “Proportion of buyers” is the sum of the first three data columns.

Of the respondents, 63% would not select poultry meat fed with GM feed (Case 3). Approximately 25% would select the GM product if it was cheaper than the conventional product. When asked, over 90% expressed the opinion that GM feed should be marked with a label.

Table 4 also reports the correlation coefficients between the purchase intentions to provide a first impression of whether the purchase intentions under various risks are correlated with each other. The correlation coefficients show stronger dependency between the intentions under the two risk types related to production, *i.e.*, chemical risk and GM risk. The purchase intention under biological risk is associated more weakly with the intentions under the other two risk types.

Table 5 presents the logistic regression models and the variables that affect the buying intention for each risk product. This provides an opportunity to compare the association of perceived risks with various background variables. For Case 1 (biological risk), the purchase intention probability was significantly affected by the amount of poultry in the respondent’s diet. This probably indicates that when the proportion of poultry in the diet increases, consumers perceive the probability of infection to increase. The effect of the health orientation of the respondent was slightly higher and had a lower *p*-value in the case of biological risk than in the models for other products. Safety orientation consistently affected the choice in all models, but the effect was slightly lower for biological risk than for the other risks. Women were more likely to reduce their purchase intention as biological risk increased, but the effect of gender was even higher for the other risk products. Younger people reacted to the biological risk more moderately than older consumers, although the *p*-value was slightly over 0.10. Domestic preference played no role in biological risks.

In the model for purchasing chemically treated poultry (Case 2), the explanatory factors differed somewhat from those in Case 1. Willingness to buy chemically treated poultry was particularly reduced by domestic preferences, indicating that it may not be the only risk attitude that is expressed in the hypothetical choice situation. The income of the respondent also played a role, with high-income respondents indicating a particularly negative intention towards buying chemically treated poultry. The effect of age was also particularly clear in the case of chemical treatment, with older people being more reluctant to buy chemically treated products.

**Table 5:** Logistic regressions for the intention to purchase risk products (dependent variable: buy = 1, not buy = 0).

Variable	Coef.	*p*-value	Coef.	*p*-value	Coef.	*p*-value
Constant	5.806	0.000	6.725	0.000	10.659	0.000
Amount of poultry in the diet	−1.304	0.000	−0.377	0.371	−0.296	0.354
Health orientation	−0.234	0.022	−0.165	0.254	−0.195	0.099
Domestic preference	−0.036	0.745	−0.889	0.000	−0.589	0.000
Safety orientation	−1.075	0.007	−1.283	0.014	−1.297	0.004
GM negativity	−0.257	0.000	−0.573	0.000	−1.500	0.000
Gender, female	−0.358	0.011	−0.568	0.011	−0.385	0.017
Age		0.317		0.109		0.074
25–34 years	−0.313	0.422	−0.427	0.295	0.044	0.901
35–54 years	−0.553	0.124	−0.862	0.024	−0.389	0.243
over 54 years	−0.574	0.111	−0.747	0.054	0.053	0.875
Gross income		0.309		0.145		0.663
€20,000–40,000	0.224	0.343	0.213	0.535	−0.145	0.589
€40,000–60,000	0.074	0.756	0.193	0.576	−0.300	0.276
€60,000–80,000	−0.15	0.568	−0.126	0.760	−0.372	0.228
over €80,000	0.302	0.341	−0.911	0.090	−0.408	0.274
N	1,226		1,226		1,226	
Proportion of buyers (data), %	70.5		11.5		37.4	
Proportion of buyers (model), %	94.5		2.7		32.0	
Chi-squared	90.60		171.72		538.63	
*p*-value	0.000		0.000		0.000	
Nagelkerke R^2^	0.10		0.26		0.49	

Note. The reference level for age is <25 years and for income <€20,000.

In Case 3, the unwillingness to buy GM-fed poultry was significantly associated with a negative attitude towards using GM feed in poultry production. Safety orientation also had a higher coefficient than in the models for the other products. As in Case 2, domestic preference also played a role, with those having preferences for domestic products being less likely to choose a GM product.

Based on the chi-squared test, all the models were acceptable, since the independent variables were significantly associated with the dependent variables. However, the pseudo R^2^ values were relatively low, particularly in the case of biological risk (0.10). The lower goodness-of-fit in this case may relate to the measured attitudes, which did not serve so well in explaining the buying intention under an increased risk of Salmonella. Nonetheless, in this case the model also revealed several significant variables associated with the purchase intention.

The possible heterogeneity of respondents regarding the purchase intentions for risk food was further analysed with the latent class logistic regression model (Table 6). The model also allowed analysis of the relative importance of risk types. Based on the model, individuals were classified into the most probable of the two groups. Approximately 60% of the respondents belonged to the first group, in which the intention to purchase risk food was significantly lower than in the second group. Among the first group there was a positive intention to buy a risky product in 22% of the cases, whereas among the second group the buying probability was 66% considering all risk products together. This lower tendency to buy risky products among the first group is visible in the negative constant in the model for risk types. In the model for risk types, the GM risk (Case 3) was used as the reference level against which the other risks were compared.

**Table 6:** Latent class logistic regression for purchase intentions. Class membership is explained with the covariates.

	Class 1:	Class 2:	*Wald*	*p-value*	*Wald(=)*	*p-value*
	Production risk avoiders	Risk neutrals				
Class share, %	59	41				
**Model for risk type**						
(Case 3 as reference)	−4.17	1.96	68.94	0.000	29.65	0.000
Constant						
Case 1	4.74	−0.65	22.38	0.000	21.98	0.000
Case 2	−0.39	−3.04	147.83	0.000	4.20	0.040
**Covariate function**						
Constant	0	10.79	41.42	0.000		
GM negativity	0	−2.10	85.84	0.000		
Domestic preference	0	−0.85	23.68	0.000		
Importance of price	0					
1 = not at all important	0	0	18.45	0.001		
2	0	0.89				
3	0	1.19				
4	0	1.63				
5 = very important	0	2.29				
Age			9.66	0.022		
25–34 years	0	−0.27				
35–54 years	0	−0.98				
over 55 years	0	−0.53				
Gender, female	0	−0.64	9.84	0.002		
Northern Finland	0	−1.18	3.97	0.046		
Overall R²		0.56				
R²	0.50	0.33				
Proportion of positive buying intentions, %	22	66				

In the first group, chemical treatment (Case 2) slightly reduced the willingness to purchase a product compared to GM risk. Compared to GM risk in this group, the increased risk of zoonoses (Case 1) had a positive effect on the probability of buying. In other words, Case 1 had less of an impact on the purchase intention than either Case 2 or Case 3 in the first group. Based on this finding, the group can be labelled as *production risk* *avoiders*. The second group experienced chemical treatment (Case 2) as the type of risk that reduces their purchasing intentions the most. Furthermore, they considered the risk in Case 1 (biological risk) to be a more important negative cue than in Case 3 (GM feed). However, as the impact of all risk types was lower than in the first group, the group was labelled as *risk neutrals*.

**Figure 1:**
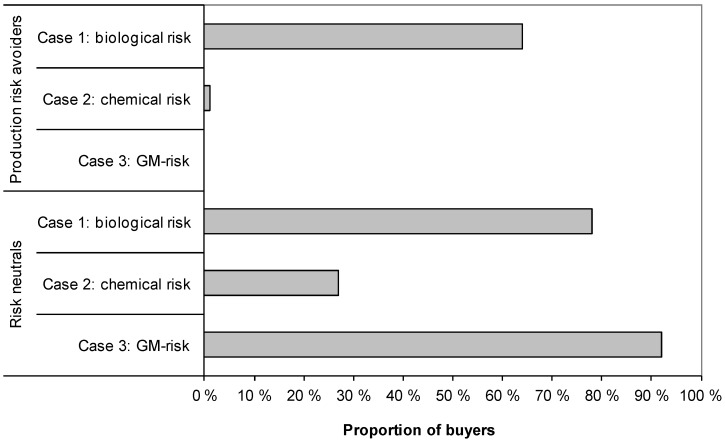
The stated intention to buy risk products in two consumer classes based on latent class logistic regression.

Figure 1 illustrates the purchase intentions in the two classes, classified with latent class regression (Table 6). It displays the class-dependent proportion of buyers for different risk products, when the overall buying proportions (70.0%, 11.1%, and 36.8% respectively, Table 4) were examined by class. The figure clearly shows the milder reaction to Case 1 in both groups. The two cases related to production divided the sample: the buying intentions for a GM product were zero per cent among the production risk avoiders, but 92% among the risk neutrals. The figure also shows that Case 2 (chemical treatment) received a strongly negative reaction in both classes. However, among the risk neutrals, 27% were still willing to buy the chemically treated products. 

The covariate function for latent classes (Table 6) confirmed the pattern given in the logistic models for each risk regarding the background variables. Table 6 describes the profile of classes, using class 1 (production risk avoiders) as the reference group. The attitude-level variables explained the buying intentions. In particular, the negative attitude towards GM food was highly important and significant in this group. They also appreciated domestic production more than the risk-neutral group. The status of price consciousness was quite natural: for the risk-neutral group, price was a more important choice criterion than for the group avoiding production risks.

The membership of latent classes could be explained by several background factors that also confirmed the results of the individual models in describing purchase intentions under increasing risk. In particular, women as well as middle-aged and older respondents belonged to the production risk-avoiding group. Considering geographical regions, only Lapland proved to be statistically significant, with a greater share of risk avoidance. The exceptionality of Lapland may be associated with greater abundance of natural products in the diet.

## 4. Discussion

The latent class model provided information on two different groups of consumers and their buying behaviour under three cases of risk. Revealing only two groups of consumers provides a somewhat simpler picture of consumer risk perceptions than the previous studies suggesting four consumer classes [30,31]. While in our study the attitude towards each risk type was negative, the proportion of those who were less sensitive to risky foods can be seen as surprisingly high, being about 40% of the consumers. This was rather close to the previous empirical results of Kennedy *et al*. [31] who—based on attitudes—provided five classes that could be simplified to those with stronger risk attitudes (58%) and those with milder attitudes (42%). The proportion of less sensitive consumers was somewhat lower in Payne *et al*. [29], in which one out of four was less sensitive to information on a new biological risk. In our study, among the less sensitive consumers were those who increased their purchasing of the product under risk if the price decreased. Similarly, for instance, Adda [26] found that some households increased their consumption of beef during the BSE epidemic. Our results also revealed that those who avoided risky poultry foods were more likely to be women, older people, and people having a large share of poultry in their diet. The greater response for women and older people has also been found in earlier studies [26,28].

Overall, the results demonstrated that safety is an important factor influencing the purchase decision in the case of poultry. The reactions to the three cases of risk (biological, chemical, technological) varied among the consumers. Measured by the purchase intention, the reaction to chemical risks was stronger than the reaction to biological risks. Case 1 (biological risk), which may to some extent be controlled by the consumer by paying attention to food preparation, seems to affect the intended purchase less than the two other cases. This is consistent with the findings in Mazzocchi *et al*. [21] and TNS Opinion and Social [22], but is nonetheless interesting, because objectively it is the only unambiguous risk of the three cases considered. The results are in contrast to those reported by Grande *et al*. [58], who found that bacteria-infected food is seen as a higher risk than chemical additives in food in Scotland as well as in Norway, and also to Yeung & Morris [18], who argued that Salmonella is rated high on the list of dread factors because of its severe consequences.

Cases 2 and 3 (chemical treatment and the use of GM feed) presented in this study are matters that rely strongly on consumers’ own perceptions and beliefs. Fear of unknown technologies [34,59] may be an important factor in the production risk avoiding group. The European Food Safety Authority (EFSA) has established that chlorine treatment of poultry meat is not a risk to human health, nor does it promote the development of resistance to antimicrobials. The avoidance of chemical risk may have more to do with domestic preference than risk *per se*. Similarly, no self-evident health impacts have been observed in relation to GM feed. The results confirm the point presented in Yeung & Morris [18] that there may be a divergence between objective technical risk assessment and subjective psychological risk assessment by the consumer. Siegrist *et al*. [60] suggested that treatments or additives that are perceived to have direct tangible benefits for consumers (such as artificial sweeteners or convenience foods) are seen as less risky than those with no obvious consumer benefits (such as GM food). Consistently with Siegrist *et al*. [60], our results may also imply that consumer benefits from a lower price are perceived differently compared to less tangible benefits.

Nonetheless, our results are consistent with the finding that the risk perceptions and risk preferences were significant determinants of the acceptance of GM food [6]. This raises the question of how to separate risk from the other negative beliefs of consumers. It may be possible to affect these experiences through the provision of objective information regarding the levels of risk, as was done for the biological risk. However, it has also been suggested that in the case of GM the perceived risks affect the purchase decisions more than the perceived benefits, and the consumers’ own knowledge concerning GM only affects the perceived risks [27]. Hence, information provision alone does not necessarily provide an adequate solution.

Beyond the type of risk, the presentation of the case (quantitative, qualitative) or the characteristics of the risk, such as voluntariness and familiarity [34], also affect purchase intentions. The microbiological risk of *Salmonella* can be seen as a voluntary hazard that is relatively familiar to the consumer. In contrast, chemical control and the use of (unlabelled) GM feed are involuntary, uncontrollable and possibly have delayed effects, and hence are rated as more risky by the consumer [18]. Given our data, the actual reasons behind the different reactions cannot be ascertained, and it would be a fruitful future topic to study the behavioural reactions towards risk products that were constructed in a controllable manner. However, not all the affecting conditions and information can be controlled. For example, in the case of our poultry data, which were collected at end of 2007, there may still have been echoes of the avian influenza news that temporarily reduced poultry consumption by 10%–15% on average in the EU [44].

The purchase intentions were statistically related to several attitude-based variables. Particularly in Case 3 (GM feed), the variation in buying probabilities was affected by the attitude. Of the attitude components used, health orientation was more strongly associated with biological risk than with production risks (chemical and GM). Production risks were more strongly driven by the other attitude components, *i.e.*, domestic preference, safety orientation and GM negativity. The strong role of attitudes follows the theory of planned behaviour [40] and previous empirical results in the case of purchase intentions for risky food. The role of the product price was also highlighted: it can be seen as a behavioural control, as suggested in the theory of planned behaviour. In the existing literature, the variable that measures the importance of price *versus* food safety has been found statistically significant [10], suggesting that the relative importance placed on safety also materialises in purchase intentions.

The information provided to the respondents in the three cases differed somewhat depending on the amount of information available. In addition to risks having different characteristics, the form of presenting the risk to people can have a substantial impact on the results, but there is not necessarily any “right” way to present the information [15,36,61]. The case of poultry meat may also have an effect on differences in buying intentions. In earlier studies, the willingness to pay to reduce foodborne illness has been found to be larger for risks transmitted through poultry than ground beef or packaged meat [28]. Additionally, this survey-based study relying on hypothetical buying intentions needs to be complemented with studies of actual behaviour. That kind of a study would also focus on the critical intention-behaviour gap found in the theory of planned behaviour applications [62,63]. However, the current comparison helps us in understanding that reactions to different kinds of risks are not always homogeneous, and different consumer segments need to be accounted for in policy design, the communication of risks and marketing strategies.

## 5. Conclusions

Based on our results, one could argue that the probability of GM-associated risks may be overestimated, whereas the biological risk of Salmonella may be underestimated by consumers. The World Trade Organization Agreement on the Application of Sanitary and Phytosanitary Measures (SPS Agreement) nonetheless requires a consistent approach towards risk in different circumstances, which may be a challenge when public responses to separate risk types differ. It also implies a need for differentiated management solutions. Our results suggest that the strong attitude-based reactions to risks in production technologies such as GM and chemical treatment demand new methods of risk management. This follows the ideas of Klinke and Renn [64], who suggest that for standard health risks, traditional risk-based management with an emphasis on scientific assessment and the reduction of exposure/probabilities is sufficient. However, for risks such as genetic modification they suggest discourse-based management with an emphasis on reaching a political consensus or agreement, the importance of procedure and transparency, the establishment of trust-generating institutions, investment in risk communication, the involvement of stakeholders, and public participation.

Our study demonstrated that food safety is a multifaceted issue, as risks are perceived differently by separate consumer groups. The sole reliance on, for instance, traditional risk-based management does not address those consumers who perceive chemical or GM risks to be large and negatively affect their utility. The provision of technical information regarding the potential risks alone does not suffice in such cases, including our production risk avoiders. An evaluation of the EU legislative framework related to GM food and feed [19] discussed the challenges related to labelling GM products. Our results suggest that it is to an extent the same people who are concerned about multiple types of production risk, and therefore it might be possible to take this into account in labelling. For instance, it might be possible that an “organically produced” label would tackle several information needs simultaneously (indicating, for instance, both “no GM feed used” and “not chemically treated”).

The results also suggest that a policy directed towards particular risks will benefit certain consumer segments. For example, a policy supporting the avoidance of GM food directs the benefits particularly to risk-avoiding consumers who base their opinion on attitudes. In contrast, a policy increasing the awareness and acceptance of GM products would benefit those consumers who are less sensitive to risks and more interested in the price of goods. A policy aiming at further control of biological risks would benefit all consumers, but the effect of the policy could be low if measured in terms of purchasing intentions. As consumers are heterogeneous, any single policy instrument is unlikely to cater for them all. For instance, information provision, precautionary approaches, and trust-building in authorities, the production process and scientific risk assessment are all needed to deal with the multiple food-related risks.

Overall, the study demonstrated that risk matters to consumers, not all risk is equal, and consumer types react somewhat differently to various types of risk. Taking such diversity into account in risk assessment, risk management and risk communication is a challenge for authorities, as well as for food manufacturers.
